# Dataset on microclimate and drone-based thermal patterns within an oil palm agroforestry system

**DOI:** 10.1016/j.dib.2021.107615

**Published:** 2021-11-24

**Authors:** Laura Somenguem Donfack, Alexander Röll, Florian Ellsäßer, Martin Ehbrecht, Bambang Irawan, Dirk Hölscher, Alexander Knohl, Holger Kreft, Eduard J. Siahaan, Leti Sundawati, Christian Stiegler, Clara Delphine Zemp

**Affiliations:** a University of Goettingen, Silviculture and Forest Ecology of the Temperate Zones, Büsgenweg 1, Göttingen 37077, Germany; b University of Goettingen, Tropical Silviculture and Forest Ecology, Büsgenweg 1, Göttingen 37077, Germany; c University of Goettingen, Biodiversity, Macroecology and Biogeography, Büsgenweg 1, Göttingen 37077, Germany; d University of Jambi, Faculty of Forestry, Jln Raya Jambi, Jambi 36361, Indonesia; e University of Goettingen, Centre of Biodiversity and Sustainable Land Use, Büsgenweg 1, Göttingen 37077, Germany; f University of Goettingen, Bioclimatology, Büsgenweg 2, Göttingen 37077 Germany; g Department of Forest Management, Bogor Agricultural University, Kampus IPB Darmaga, Bogor 16680, Indonesia; h University of Neuchâtel, Institute of Biology, Conservation Biology Lab, Rue Emilie-Argand 11, Neuchâtel CH-2000, Switzerland

**Keywords:** Microclimate, Land surface temperature, iButton, Drone-based thermography, Oil palm, Agroforestry, Vegetation structure, Biodiversity

## Abstract

Microclimate and Land Surface Temperature (LST) are important analytical variables used to understand complex oil palm agroforestry systems and their effects on biodiversity and ecosystem functions. In order to examine experimental effects of tree species richness (0, 1, 2, 3 or 6), plot size (25 m^2^, 100 m^2^, 400 m^2^, 1600 m^2^) and stand structural complexity on microclimate and Land Surface Temperature, related data were collected following a strict design. The experiment was carried out in the Jambi province, in Sumatra (Indonesia), as part of the collaborative project EFForTS [Ecological and Socioeconomic Functions of Tropical Lowland Rainforest Transformation Systems]. Microclimate data collected using miniaturized data loggers combined with drone-based thermal data were considered within an oil palm plantation enriched with six target tree species. The timeframe considered for data analysis was 20th September 2017 to 26th September 2017. The experiment data can be used for comparison with data from conventional oil palm agroforestry systems in the tropics. They can more specifically be used as reference to assess microclimate and Land Surface Temperature patterns within similar agroforestry systems.

## Specifications Table

**Table:** 

Subject	Ecology
Specific subject area	Assessment of microclimate and land surface temperature variability within oil palm plots enriched with tree species.
Type of data	Table Image Figure
How data were acquired	Data were obtained using miniaturized microclimate sensors (hygrochron and thermochron loggers), and an octocopter drone equipped with radiometric thermal and RGB (red-green-blue) cameras.
Data format	Raw Analyzed
Parameters for data collection	Ambient air temperature, soil temperature, relative humidity and land surface temperature data acquired over a week (20^th^ September 2017 to 26^th^ September 2017) and around noon were considered.
Description of data collection	Microclimate data were collected from 28 experimental plots and 4 control plots varying in size (25 m^2^, 100 m^2^, 400 m^2^ and 1600 m^2^) and in diversity level (0, 1, 2, 3 and 6). Land surface temperature data were obtained from 52 experimental plots and 4 control plots.
Data source location	Institution: EFForTS [Ecological and Socioeconomic Functions of Tropical Lowland Rainforest Transformation Systems] City: Jambi (Sumatra) Country: Indonesia Latitude and longitude (and GPS coordinates, if possible) for collected samples/data: 01.95° S and 103.25° E
Data accessibility	With the article (Supplementary part) Repository name: Mendeley Data Data identification number: https://doi.org/10.17632/79t4psrhwj.1 Direct URL to data: https://data.mendeley.com/datasets/79t4psrhwj/3
Related research article	L.S. Donfack, A. Röll, F. Ellsäßer, M. Ehbrecht, B. Irawan, D. Hölscher, A. Knohl, H. Kreft, E.J. Siahaan, L. Sundawati, C. Stiegler, D. C. Zemp, Microclimate and land surface temperature in a biodiversity enriched oil palm plantation, Forest Ecology and Management. https://doi.org/10.1016/j.foreco.2021.119480

## Value of the Data


 

• This dataset provides valuable spatial and temporal temperature and humidity data obtained from permanent plots established in an agroforest stand. It might be useful to understand the effect of mixed species tree planting on microclimate and land surface temperature variability.
• These data will benefit those who are interested in agroforestry management systems, biodiversity and ecosystem functions enhancement and efficient techniques for microclimate data collection and assessment.
• The data can be used to be compared with other microclimate and land surface temperature data collected in tropical agroforestry systems or pure oil palm plantations. They can also be used to assess effect of other parameters (e.g., vapour pressure deficit, tree growth, transpiration, etc.) on thermal patterns.

## 1 Data Description

### 1.1 Microclimate data

Ambient air temperature, soil temperature and relative humidity represent the microclimate data of interest recorded. The dataset containing microclimate data is structured with text files categorized with *date, Time, values (temperatures/relative humidity values) and Unit*. These are raw data extracted from microclimate sensors.

### 1.2 Land surface temperature data

Land Surface Temperatures were measured at the surface of oil palm and tree canopy. Thermal images from 56 plots and a *csv* file summarizing climatic variables for these plots and their metrics (average, minimum, maximum etc.) are available in the data repository. Thermal images were stored as *tif* images that can be visualized in GIS software (e.g. QGIS) and their respective properties can also be checked there. Abbreviations used in the csv file named “*AllData_Humusindo_56Plots_stats.csv*” are summarized (Table. 1);

**Table 1:** List of abbreviations in the File “*AllData_Humusindo_56Plots_stats.csv*”.

name	typeOfVariable	units	description
Date	date		date and time
Pressure_hPa	realNumber	hPa	air pressure
Q_Pressure	integerNumber		quality flag air pressure
Min_Pressure_hPa	realNumber	hPa	minimum air pressure
Q_Min_Pressure	integerNumber		quality flag minimum air pressure
Max_Pressure_hPa	realNumber	hPa	maximum air pressure
Q_Max_Pressure	integerNumber		quality flag maximum air pressure
PrTemp_degC	realNumber	°C	pressure temperature
Q_PrTemp	integerNumber		quality flag pressure temperature
Min_PrTemp_degC	realNumber	°C	minimum pressure temperature
Q_Min_PrTemp	integerNumber		quality flag minimum pressure temperature
Max_PrTemp_degC	realNumber	°C	maximum pressure temperature
Q_Max_PrTemp	integerNumber		quality flag maximum pressure temperature
UBat_var_V	realNumber	V	battery voltage
Q_UBat_var	integerNumber		quality flag battery voltage
Min_UBat_var_V	realNumber	V	minimum battery voltage
Q_Min_UBat_var	integerNumber		quality flag minimum battery voltage
Max_UBat_var_V	realNumber	V	maximum battery voltage
Q_Max_UBat_var	integerNumber		quality flag maximum battery voltage
CMP3_Radiation_W_per_m	realNumber	W/m^2^	Global radiation
Q_CMP3_Radiation	integerNumber		quality flag global radiation
Min_CMP3_Radiation_W_per_m	realNumber	W/m^2^	minimum global radiation
Q_Min_CMP3_Radiation	integerNumber		quality flag minimum global radiation
Max_CMP3_Radiation_W_per_m	realNumber	W/m^2^	maximum global radiation
Q_Max_CMP3_Radiation	integerNumber		quality flag maximum global radiation
NR_Radiation_W_per_m	realNumber	W/m^2^	net radiation
Q_NR_Radiation	integerNumber		quality flag net radiation
Min_NR_Radiation_W_per_m	realNumber	W/m^2^	minimum net radiation
Q_Min_NR_Radiation	integerNumber		quality flag minimum net radiation
Max_NR_Radiation_W_per_m	realNumber	W/m^2^	maximum net radiation
2_Max_NR_Radiation	integerNumber		quality flag maximum net radiation
PAR_Quantum_mol_per_ms	realNumber	umol/m^2^s	incoming PAR
Q_PAR_Quantum	integerNumber		quality flag PAR
Min_PAR_Quantum_mol_per_ms	realNumber	mol/m^2^s	minimum PAR
Q_Min_PAR_Quantum	integerNumber		quality flag minimum PAR
Max_PAR_Quantum_mol_per_ms	realNumber	mol/m^2^s	maximum PAR
Q_Max_PAR_Quantum	integerNumber		quality flag maximum PAR
WS_FC_m_per_S	realNumber	m/s	wind speed
Q_WS_FC	integerNumber		quality flag wind speed
Min_WS_FC_m_per_s	realNumber	m/s	minimum wind speed
Q_Min_WS_FC	integerNumber		quality flag minimum wind speed
Max_WS_FC_m_per_s	realNumber	m/s	maximum wind speed
Q_Max_WS_FC	integerNumber		quality flag maximum wind speed
WD_FC_deg	realNumber	o	wind direction
Q_WD_FC	integerNumber		quality flag wind direction
Min_WD_FC_deg	realNumber	o	minimum wind direction
Q_Min_WD_FC	integerNumber		quality flag minimum wind direction
Max_WD_FC_deg	realNumber	o	maximum wind direction
Q_Max_WD_FC	integerNumber		quality flag maximum wind direction
Humidity1_prc	realNumber	Vol.%	air humidity, 0.5 m
Q_Humidity1	integerNumber		quality flag air humidity 0.5 m
Min_Humidity1_pro	realNumber	Vol.%	minimum air humidity, 0.5 m
Q_Min_Humidity1	integerNumber		quality flag minimum air humidity 0.5 m
Max_Humidity1_pro	realNumber	Vol.%	maximum air humidity, 0.5 m
Q_Max_Humidity1	integerNumber		quality flag maximum air humidity 0.5 m
Temperature1_degC	realNumber	°C	air temperature, 0.5 m
Q_Temperature1	integerNumber		quality flag air temperature, 0.5 m
Min_Temperature1_degC	realNumber	°C	minimum air temperature, 0.5 m
2_Min_Temperature1	integerNumber		quality flag minimum air temperature, 0.5 m
Max_Temperaturel_degC	realNumber	°C	maximum air temperature, 0.5 m
Q_Max_Temperature1	integerNumber		quality flag maximum air temperature, 0.5 m
Humidity2_pro	realNumber	Vol.%	air humidity, 2 m
Q_Humidity2	integerNumber		quality flag air humidity 2 m
Min_Humidity2_prc	realNumber	Vol.%	minimum air humidity, 2 m
Q_Min_Humidity2	integerNumber		quality flag minimum air humidity 2 m
Max_Humidity2_pro	realNumber	Vol.%	maximum air humidity, 2 m
Q_Max_Humidity2	integerNumber		quality flag maximum air humidity 2 m
Temperature2_degC	realNumber	°C	air temperature, 2 m
Q_Temperature2	integerNumber		quality flag air temperature, 2 m
Min_Temperature2_degC	realNumber	°C	minimum air temperature, 2 m
Q_Min_Temperature2	integerNumber		quality flag minimum air temperature, 2 m
Max_Temperature2_degC	realNumber	°C	maximum air temperature, 2 m
Q_Max_Temperature2	integerNumber		quality flag maximum air temperature, 2 m
HeatFluxPlate_W_per_m	realNumber	W/m^2^	heat flux plate
Q_HeatFluxPlate	integerNumber		quality flag heat flux plate
Min_HeatFluxPlate_W_per_m	realNumber	W/m^2^	minimum heat flux plate
Q_Min_HeatFluxPlate	integerNumber		quality flag minimum heat flux plate
Max_HeatFluxPlate_W_per_	realNumber	W/m^2^	maximum heat flux plate
Q_Max_HeatFluxPlate	integerNumber		quality flag maximum heat flux plate
Precipitation1_mm	realNumber	mm	precipitation1
Q_Precipitation1	integerNumber		quality flag precipitation1
Precipitation2_mm	realNumber	mm	precipitation2
Q_Precipitation2	integerNumber		quality flag precipitation2
Moisture1_Vol_prc	realNumber	Vol.%	soil moisture 1, 30 cm
Q_Moisture1	integerNumber		quality flag soil moisture 1, 30 cm
Min_Moisture1_Vol_pre	realNumber	Vol.%	minimum soil moisture 1, 30 cm
Q_Min_Moisture1	integerNumber		quality flag minimum soil moisture 1, 30 cm
Max_Moisture1_Vol_prc	realNumber	Vol.%	maximum soil moisture 1, 30 cm
Q_Max_Moisture1	integerNumber		quality flag maximum soil moisture 1, 30 cm
SoilTemp1_degC	realNumber	°C	soil temperature 1, 30 cm
Q_SoilTemp1	integerNumber		quality flag soil temperature 1, 30 cm
Min_SoilTemp1_degC	realNumber	°C	minimum soil temperature 1, 30 cm
Q_Min_SoilTemp1	integerNumber		quality flag minimum soil temperature 1, 30 cm
Max_SoilTemp1_degC	realNumber	°C	maximum soil temperature 1, 30 cm
Q_Max_SoilTemp1	integerNumber		quality flag maximum soil temperature 1, 30 cm
Moisture2_Vol_prc	realNumber	Vol.%	soil moisture 2, 30 cm
Q_Moisture2	integerNumber		quality flag soil moisture 2, 30 cm
Min_Moisture2_Vol_prc	realNumber	Vol.%	minimum soil moisture 2, 30 cm
Q_Min_Moisture2	integerNumber		quality flag minimum soil moisture 2, 30 cm
Max_Moisture2_Vol_pro	realNumber	Vol.%	maximum soil moisture 2, 30 cm
Q_Max_Moisture2	integerNumber		quality flag maximum soil moisture 2, 30 cm
SoilTemp2_degC	realNumber	°C	soil temperature 2, 30 cm
Q_SoilTemp2	integerNumber		quality flag soil temperature 2, 30 cm
Min_SoilTemp2_degC	realNumber	°C	minimum soil temperature 2, 30 cm
Q_Min_SoilTemp2	integerNumber		quality flag minimum soil temperature 2, 30 cm
Max_SoilTemp2_degC	realNumber	°C	maximum soil temperature 2, 30 cm
Q_Max_SoilTemp2	integerNumber		quality flag maximum soil temperature 2, 30 cm
Moisture3_Vol_prc	realNumber	Vol.%	soil moisture 3, 30 cm
Q_Moisture3	integerNumber		quality flag soil moisture 3, 30 cm
Min_Moisture3_Vol_pro	realNumber	Vol.%	minimum soil moisture 3, 30 cm
Q_Min_Moisture3	integerNumber		quality flag minimum soil moisture 3, 30 cm
Max_Moisture3_Vol_prc	realNumber	Vol.%	maximum soil moisture 3, 30 cm
Q_Max_Moisture3	integerNumber		quality flag maximum soil moisture 3, 30 cm
SoilTemp3_degC	realNumber	°C	soil temperature 3, 30 cm
Q_SoilTemp3	integerNumber		quality flag soil temperature 3, 30 cm
Min_SoilTemp3_degC	realNumber	°C	minimum soil temperature 3, 30 cm
Q_Min_SoilTemp3	integerNumber		quality flag minimum soil temperature 3, 30 cm
Max_SoilTemp3_degC	realNumber	°C	maximum soil temperature 3, 30 cm
Q_Max_SoilTemp3	integerNumber		quality flag maximum soil temperature 3, 30 cm

### 1.3 Other abbreviations used in the dataset

RH: relative humidity; LST: land surface temperature

### 1.4 Appendices

Raw data available in the Mendeley repository include a first folder containing appendices summarized in a *docx* file. Appendix A contains a detailed enumeration and denomination of microclimate sensors positioned above and under the ground, in the respective plots within which they were found. It shows which sensors successfully collected data and which didn't. Appendix B display metrics (mean, maximum, minimum, median and standard deviation) of microclimate (for 32 plots) and land surface temperature (for 56 plots) calculated considering the timeframe 10 am to 3 pm. Appendix C summarizes the proportion of microclimate sensors with available data for each investigated plot. Appendix D makes a state of all plots and categories of plot size and diversity level within which they are positioned. Appendices E and F show calculated values of mean, median, standard error of the mean, minimum/maximum values of ambient air, soil temperatures, relative humidity and land surface temperature, considering plot size categories and species diversity levels respectively. Appendix G contains two tables (table G.1 and G.2), displaying calibration results of temperature and relative humidity variables. To support these statistics, appendix J presents an illustrative figure of the distance effect on microclimate variables. Appendix H summarizes statistics explaining the effect of distance variation from plot centre on microclimatic values. Appendix I contains two figures showing microclimate patterns over the period of data acquisition, for microclimate variables ambient temperature, relative humidity and soil temperature. Appendix K illustrates the specific design of miniaturized microclimate sensors disposition within varying-sized plots. Appendix L presents an illustrative image of field installations (sensors and hand-made protection shields).

## 2 Experimental Design, Materials and Methods

Microclimate data were obtained from 28 plots systematically varying in size and tree species diversity, while land surface temperature data were collected from 52 plots. Microclimate data represent environmental variables recorded above (ambient air temperature and relative humidity) and below (soil temperature) the ground. Four additional control plots differently considered for microclimate and land surface temperatures data were also investigated. 198 miniaturized microclimate sensors (100 thermochron and 98 hygrochron iButtons, Maxim integrated, USA) to measure ambient air temperature, relative humidity, and soil temperature, which were acquired in 10-minutes intervals all over considered days. Due to a limited number of available sensors, we selected from the 56 initial plots, 32 that represented a great variability in vegetation structure and with varying species diversity level and plot size, including the four control plots.

**Fig. 1:**
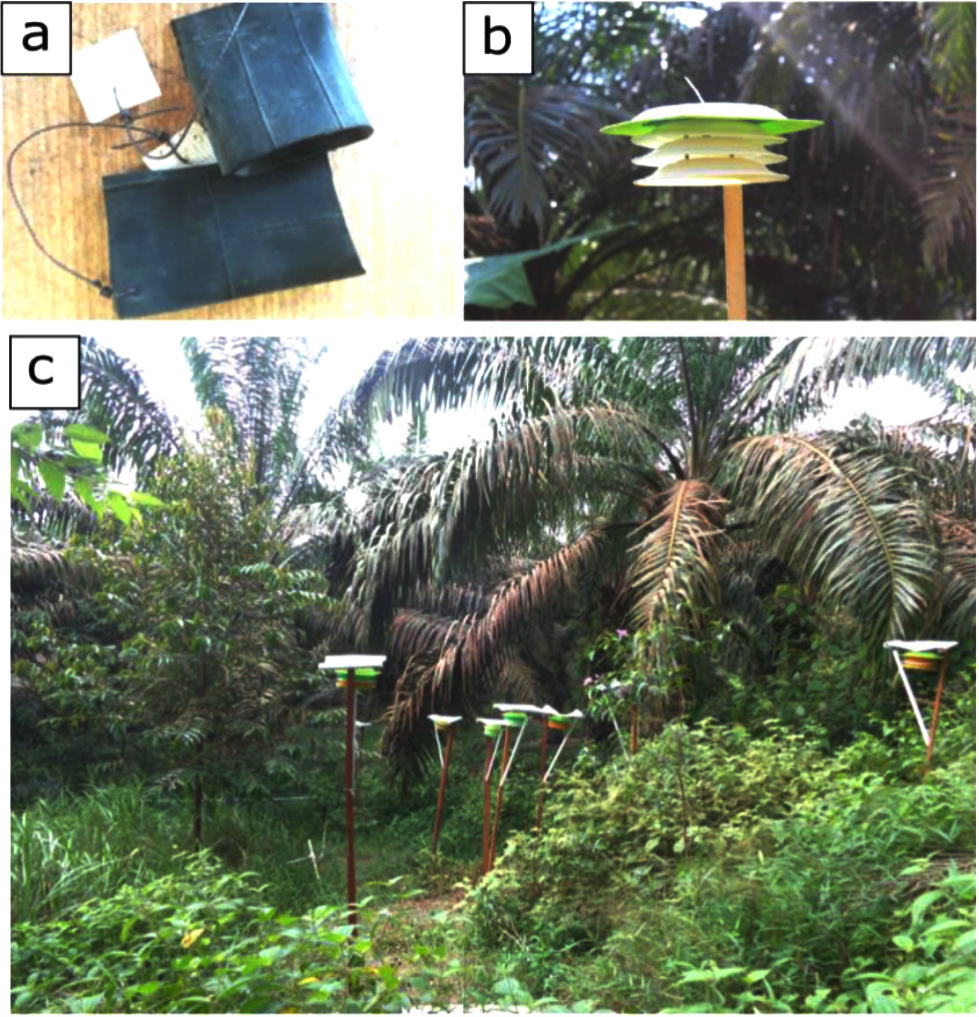
Installation of the mini microclimate sensors. (a) Protection with hand-made rubber envelope before burying the sensor below ground for measuring soil temperature; (b) Protection with hand-made shield for the sensors measuring air temperature and humidity. (c) Sensors installed in an experimental plot.

Within each plot, mini microclimate sensors were positioned on each sampling point located at increasing distance on a logarithmic scale (1, 2, 4 and 8 m distant from each other) oriented along three main directions: North, South-East, and South-West. The purpose of this fractal design was to account for spatial variations in temperature and humidity values [1] and to have comparable data across plot sizes. We used two types of mini microclimate sensors: hygrochron temperature/humidity loggers (DS1923-F5#), installed 1.5 m above the ground to measure both the ambient air temperature and relative humidity and thermochron temperature loggers (DS1922L-F5#), placed 10 cm under the ground to measure soil temperature. The mini microclimate sensors were protected from water and direct solar radiations using hand-made multi-plate radiation shields (Fig. 1).

Precision and accuracy as provided by the manufacturer are 0.063°C and 0.5°C, respectively for hygrochron and thermochron loggers. When measured values were negative, these were considered as aberrant and therefore excluded from the analysis to prevent any bias. We validated the mini microclimate sensors by applying a linear regression of the measured values with reference values (air temperature and relative humidity measured with Hygro-Thermo Transmitter, Thies Clima, Göttingen, Germany; soil temperature measured with Trime-Pico32, IMKO, Ettingen, Germany) across a range of controlled microclimatic conditions (from 25 to 35°C), indicating no systematic biases (mean slope = 0.86, mean intercept = 5.68°C, mean R^2^ = 0.91 for temperature, mean slope = 0.96, mean intercept = 3.56%, mean R^2^ = 0.91 for relative humidity). Collected data were saved as txt. files.

Thermal images were acquired with an octocopter drone (MK EASY Okto V3; HiSystems, Moormerland, Germany) equipped with radiometric thermal and RGB (red-green-blue) cameras, to capture thermal and RGB images of all the 56 study plots. We used the thermal camera Flir Tau 2 640 with TeAx ThermoCapture module attached; the focal length 13 mm covers spectral bands ranging from 7.5 to 13.5 µm. RGB camera was based on an Omni vision OV12890 CMOS-Sensor 148 (Omni vision, USA) with a 170 °FOV fish-eye lens [2]. For each day considered, flights were operated at noon (12 pm local time) at the average height of 50 m above the starting point, but varying up to 20 m over plots. Thermal images were recorded and only images falling within plot dimensions were cropped and saved as rgb *tif* files. The resulting plot thermal images were used to compute minimum, maximum and average values of temperature in degree Kelvin per plot, using thermal pixels. Thermal values per plot were saved as csv file.

We derived the stand structural complexity from terrestrial laser scans in October and November 2016 based on a procedure described by Ehbrecht et al. (2021) [3]. A FARO Focus terrestrial laser scanner (Faro Technologies Inc., Lake Mary, USA), placed at the centre of each plot was used to obtain three-dimensional point clouds of each plot for the computation of the Stand Structural Complexity Index (SSCI). SSCI is an integrated measure of the three-dimensional arrangement of the vegetation above the herbaceous layer and quantifies the heterogeneity of biomass distribution in three-dimensional space [4]. Index values increase with increasing efficiency of canopy space occupation and vertical stratification. Further details on SSCI construction and functioning can be found in Ehbrecht et al. (2021). Control plots considered to assess stand structural complexity, Land Surface Temperature (LST) and microclimate were all different, and correlations between the three variable categories were therefore performed considering 28 plots instead of 32.

We used R version 3.6.3 to calculate different metrics (mean, median, maximum/minimum, standard error of the mean and range) of each variable (ambient air temperature, relative humidity, soil temperature and LST). Microclimate metrics calculation took into consideration microclimate sensors at equivalent distance away from central sensor (1 meters) for all plots, so as to cancel the distance effect.

## Ethics Statement

None

## CRediT authorship contribution statement

**Laura Somenguem Donfack:** Conceptualization, Methodology, Data curation, Formal analysis, Writing – original draft. **Alexander Röll:** Writing – review & editing, Supervision. **Florian Ellsäßer:** Methodology, Investigation, Software, Writing – review & editing. **Martin Ehbrecht:** Methodology, Writing – review & editing. **Bambang Irawan:** Project administration, Methodology. **Dirk Hölscher:** Conceptualization, Funding acquisition, Methodology, Writing – review & editing. **Alexander Knohl:** Data curation, Writing – review & editing. **Holger Kreft:** Conceptualization, Funding acquisition, Methodology, Writing – review & editing. **Eduard J. Siahaan:** Methodology, Data curation. **Leti Sundawati:** Project administration, Methodology. **Christian Stiegler:** Methodology, Writing – review & editing. **Clara Delphine Zemp:** Conceptualization, Funding acquisition, Methodology, Investigation, Visualization, Writing – review & editing, Supervision.

## Declaration of Competing Interest

The authors declare that they have no known competing financial interests or personal relationships which have or could be perceived to have influenced the work reported in this article.
